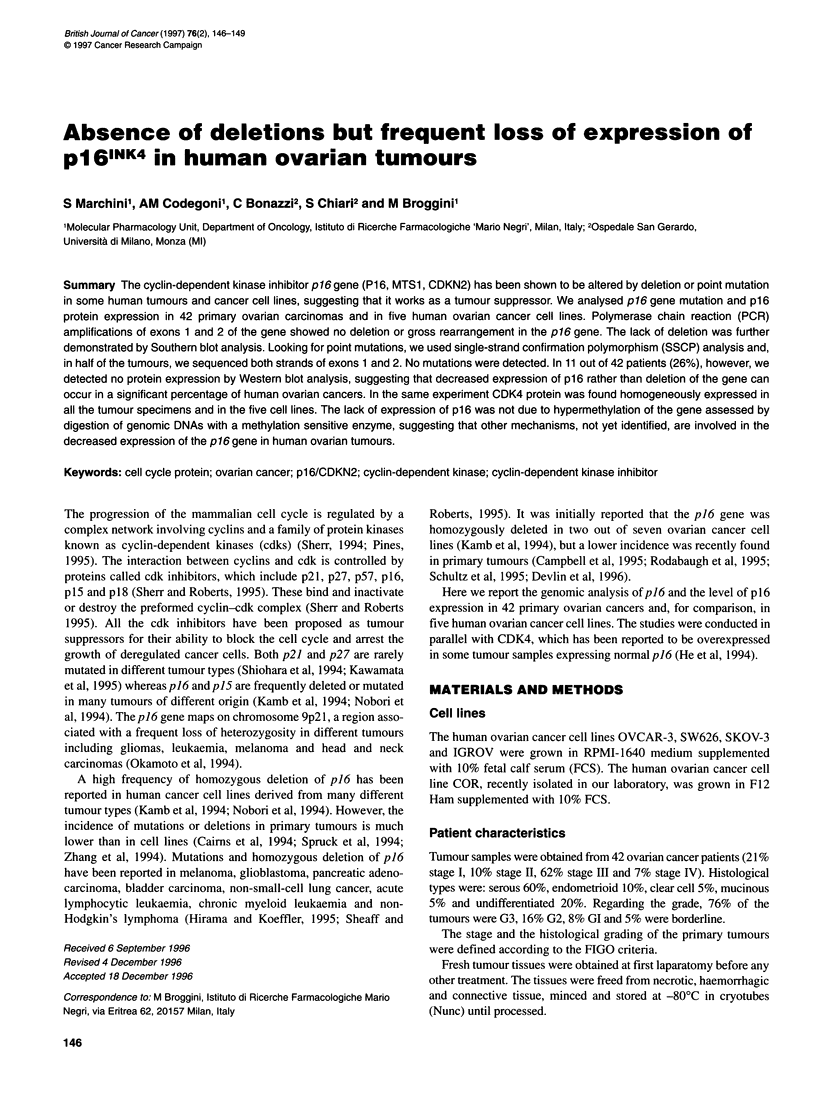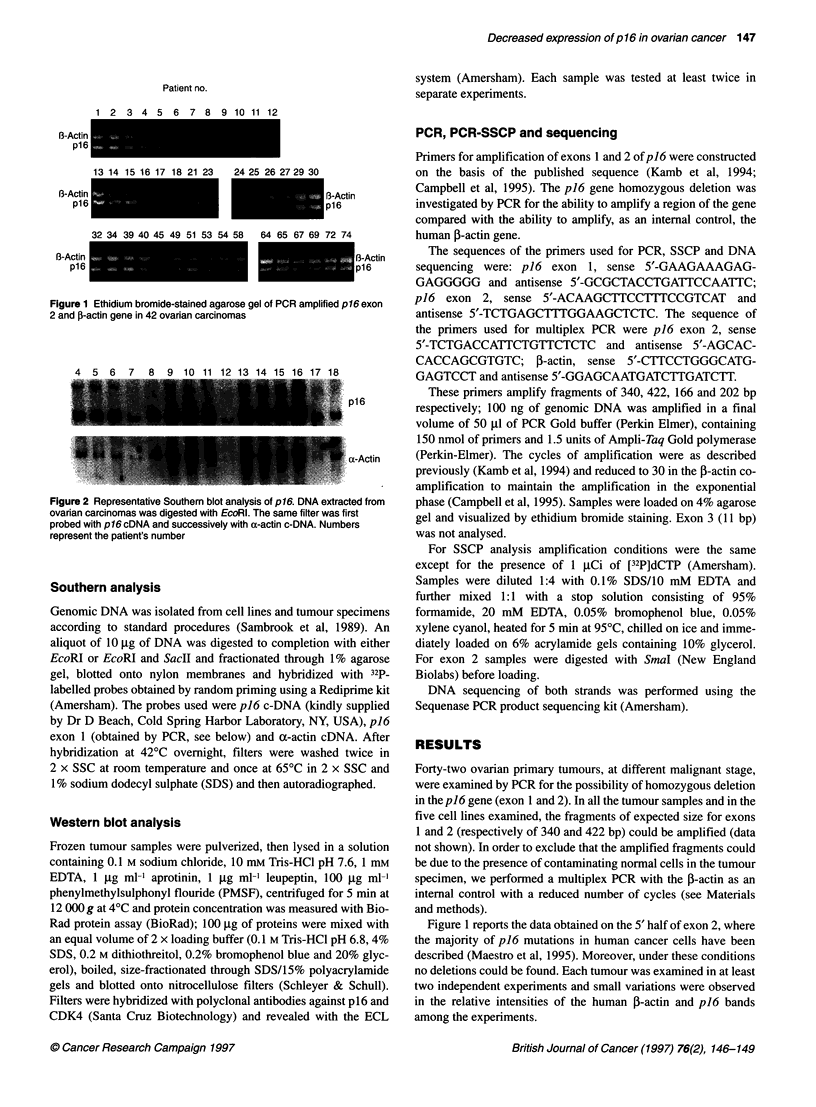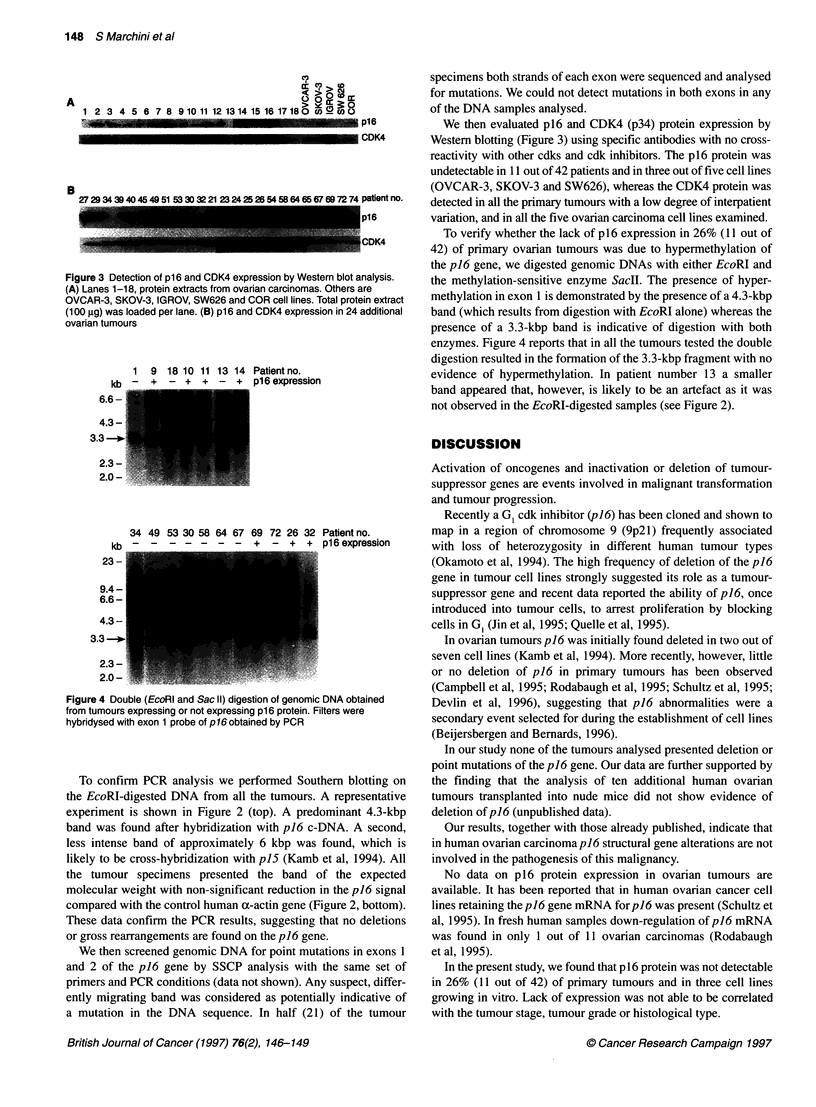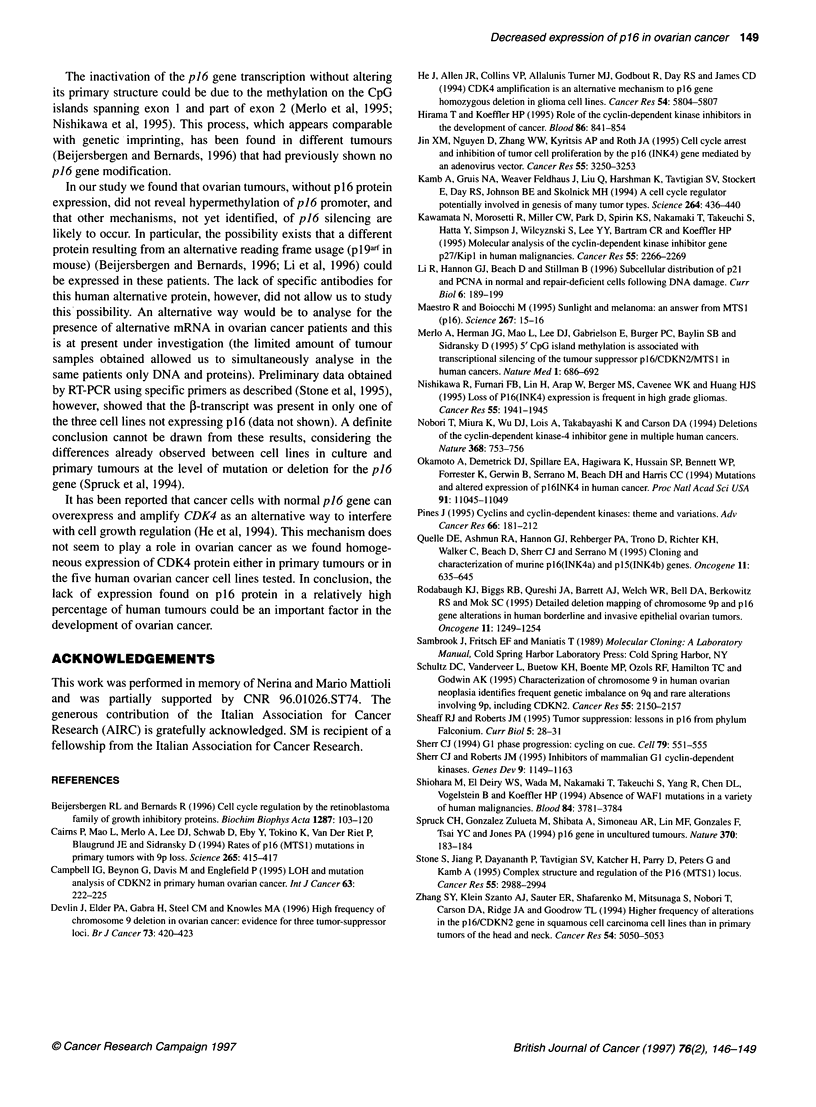# Absence of deletions but frequent loss of expression of p16INK4 in human ovarian tumours.

**DOI:** 10.1038/bjc.1997.355

**Published:** 1997

**Authors:** S. Marchini, A. M. Codegoni, C. Bonazzi, S. Chiari, M. Broggini

**Affiliations:** Department of Oncology, Istituto di Ricerche Farmacologiche Mario Negri, Milan, Italy.

## Abstract

**Images:**


British Journal of Cancer (1997) 76(2), 146–149
© 1997 Cancer Research Campaign

### Absence of deletions but frequent loss of expression of p16$^{INK4}$ in human ovarian tumours

S Marchini[1], AM Codegoni[1], C Bonazzi[2], S Chiari[2] and M Broggini[1]

[1]Molecular Pharmacology Unit, Department of Oncology, Istituto di Ricerche Farmacologiche 'Mario Negri', Milan, Italy; [2]Ospedale San Gerardo, Università di Milano, Monza (MI)

**Summary** The cyclin-dependent kinase inhibitor *p16* gene (P16, MTS1, CDKN2) has been shown to be altered by deletion or point mutation in some human tumours and cancer cell lines, suggesting that it works as a tumour suppressor. We analysed *p16* gene mutation and p16 protein expression in 42 primary ovarian carcinomas and in five human ovarian cancer cell lines. Polymerase chain reaction (PCR) amplifications of exons 1 and 2 of the gene showed no deletion or gross rearrangement in the *p16* gene. The lack of deletion was further demonstrated by Southern blot analysis. Looking for point mutations, we used single-strand confirmation polymorphism (SSCP) analysis and, in half of the tumours, we sequenced both strands of exons 1 and 2. No mutations were detected. In 11 out of 42 patients (26%), however, we detected no protein expression by Western blot analysis, suggesting that decreased expression of p16 rather than deletion of the gene can occur in a significant percentage of human ovarian cancers. In the same experiment CDK4 protein was found homogeneously expressed in all the tumour specimens and in the five cell lines. The lack of expression of p16 was not due to hypermethylation of the gene assessed by digestion of genomic DNAs with a methylation sensitive enzyme, suggesting that other mechanisms, not yet identified, are involved in the decreased expression of the *p16* gene in human ovarian tumours.

**Keywords:** cell cycle protein; ovarian cancer; p16/CDKN2; cyclin-dependent kinase; cyclin-dependent kinase inhibitor

The progression of the mammalian cell cycle is regulated by a complex network involving cyclins and a family of protein kinases known as cyclin-dependent kinases (cdks) (Sherr, 1994; Pines, 1995). The interaction between cyclins and cdk is controlled by proteins called cdk inhibitors, which include p21, p27, p57, p16, p15 and p18 (Sherr and Roberts, 1995). These bind and inactivate or destroy the preformed cyclin–cdk complex (Sherr and Roberts 1995). All the cdk inhibitors have been proposed as tumour suppressors for their ability to block the cell cycle and arrest the growth of deregulated cancer cells. Both *p21* and *p27* are rarely mutated in different tumour types (Shiohara et al, 1994; Kawamata et al, 1995) whereas *p16* and *p15* are frequently deleted or mutated in many tumours of different origin (Kamb et al, 1994; Nobori et al, 1994). The *p16* gene maps on chromosome 9p21, a region associated with a frequent loss of heterozygosity in different tumours including gliomas, leukaemia, melanoma and head and neck carcinomas (Okamoto et al, 1994).

A high frequency of homozygous deletion of *p16* has been reported in human cancer cell lines derived from many different tumour types (Kamb et al, 1994; Nobori et al, 1994). However, the incidence of mutations or deletions in primary tumours is much lower than in cell lines (Cairns et al, 1994; Spruck et al, 1994; Zhang et al, 1994). Mutations and homozygous deletion of *p16* have been reported in melanoma, glioblastoma, pancreatic adenocarcinoma, bladder carcinoma, non-small-cell lung cancer, acute lymphocytic leukaemia, chronic myeloid leukaemia and non-Hodgkin's lymphoma (Hirama and Koeffler, 1995; Sheaff and Roberts, 1995). It was initially reported that the *p16* gene was homozygously deleted in two out of seven ovarian cancer cell lines (Kamb et al, 1994), but a lower incidence was recently found in primary tumours (Campbell et al, 1995; Rodabaugh et al, 1995; Schultz et al, 1995; Devlin et al, 1996).

Here we report the genomic analysis of *p16* and the level of p16 expression in 42 primary ovarian cancers and, for comparison, in five human ovarian cancer cell lines. The studies were conducted in parallel with CDK4, which has been reported to be overexpressed in some tumour samples expressing normal *p16* (He et al, 1994).

### MATERIALS AND METHODS

### Cell lines

The human ovarian cancer cell lines OVCAR-3, SW626, SKOV-3 and IGROV were grown in RPMI-1640 medium supplemented with 10% fetal calf serum (FCS). The human ovarian cancer cell line COR, recently isolated in our laboratory, was grown in F12 Ham supplemented with 10% FCS.

### Patient characteristics

Tumour samples were obtained from 42 ovarian cancer patients (21% stage I, 10% stage II, 62% stage III and 7% stage IV). Histological types were: serous 60%, endometrioid 10%, clear cell 5%, mucinous 5% and undifferentiated 20%. Regarding the grade, 76% of the tumours were G3, 16% G2, 8% GI and 5% were borderline.

The stage and the histological grading of the primary tumours were defined according to the FIGO criteria.

Fresh tumour tissues were obtained at first laparatomy before any other treatment. The tissues were freed from necrotic, haemorrhagic and connective tissue, minced and stored at –80°C in cryotubes (Nunc) until processed.

*Received 6 September 1996*
*Revised 4 December 1996*
*Accepted 18 December 1996*

*Correspondence to:* M Broggini, Istituto di Ricerche Farmacologiche Mario Negri, via Eritrea 62, 20157 Milan, Italy

**Figure 1** Ethidium bromide-stained agarose gel of PCR amplified *p16* exon 2 and β-actin gene in 42 ovarian carcinomas

**Figure 2** Representative Southern blot analysis of *p16*. DNA extracted from ovarian carcinomas was digested with *Eco*RI. The same filter was first probed with *p16* cDNA and successively with α-actin c-DNA. Numbers represent the patient's number

### Southern analysis

Genomic DNA was isolated from cell lines and tumour specimens according to standard procedures (Sambrook et al, 1989). An aliquot of 10 μg of DNA was digested to completion with either *Eco*RI or *Eco*RI and *Sac*II and fractionated through 1% agarose gel, blotted onto nylon membranes and hybridized with $^{32}P$-labelled probes obtained by random priming using a Rediprime kit (Amersham). The probes used were *p16* c-DNA (kindly supplied by Dr D Beach, Cold Spring Harbor Laboratory, NY, USA), *p16* exon 1 (obtained by PCR, see below) and α-actin cDNA. After hybridization at 42°C overnight, filters were washed twice in 2 × SSC at room temperature and once at 65°C in 2 × SSC and 1% sodium dodecyl sulphate (SDS) and then autoradiographed.

### Western blot analysis

Frozen tumour samples were pulverized, then lysed in a solution containing 0.1 M sodium chloride, 10 mM Tris-HCl pH 7.6, 1 mM EDTA, 1 μg $ml^{-1}$ aprotinin, 1 μg $ml^{-1}$ leupeptin, 100 μg $ml^{-1}$ phenylmethylsulphonyl flouride (PMSF), centrifuged for 5 min at 12 000 *g* at 4°C and protein concentration was measured with Bio-Rad protein assay (BioRad); 100 μg of proteins were mixed with an equal volume of 2 × loading buffer (0.1 M Tris-HCl pH 6.8, 4% SDS, 0.2 M dithiothreitol, 0.2% bromophenol blue and 20% glycerol), boiled, size-fractionated through SDS/15% polyacrylamide gels and blotted onto nitrocellulose filters (Schleyer & Schull). Filters were hybridized with polyclonal antibodies against p16 and CDK4 (Santa Cruz Biotechnology) and revealed with the ECL system (Amersham). Each sample was tested at least twice in separate experiments.

### PCR, PCR-SSCP and sequencing

Primers for amplification of exons 1 and 2 of *p16* were constructed on the basis of the published sequence (Kamb et al, 1994; Campbell et al, 1995). The *p16* gene homozygous deletion was investigated by PCR for the ability to amplify a region of the gene compared with the ability to amplify, as an internal control, the human β-actin gene.

The sequences of the primers used for PCR, SSCP and DNA sequencing were: *p16* exon 1, sense 5′-GAAGAAAGAG-GAGGGGG and antisense 5′-GCGCTACCTGATTCCAATTC; *p16* exon 2, sense 5′-ACAAGCTTCCTTTCCGTCAT and antisense 5′-TCTGAGCTTTGGAAGCTCTC. The sequence of the primers used for multiplex PCR were *p16* exon 2, sense 5′-TCTGACCATTCTGTTCTCTC and antisense 5′-AGCAC-CACCAGCGTGTC; β-actin, sense 5′-CTTCCTGGGCATG-GAGTCCT and antisense 5′-GGAGCAATGATCTTGATCTT.

These primers amplify fragments of 340, 422, 166 and 202 bp respectively; 100 ng of genomic DNA was amplified in a final volume of 50 μl of PCR Gold buffer (Perkin Elmer), containing 150 nmol of primers and 1.5 units of Ampli-*Taq* Gold polymerase (Perkin-Elmer). The cycles of amplification were as described previously (Kamb et al, 1994) and reduced to 30 in the β-actin co-amplification to maintain the amplification in the exponential phase (Campbell et al, 1995). Samples were loaded on 4% agarose gel and visualized by ethidium bromide staining. Exon 3 (11 bp) was not analysed.

For SSCP analysis amplification conditions were the same except for the presence of 1 μCi of [$^{32}P$]dCTP (Amersham). Samples were diluted 1:4 with 0.1% SDS/10 mM EDTA and further mixed 1:1 with a stop solution consisting of 95% formamide, 20 mM EDTA, 0.05% bromophenol blue, 0.05% xylene cyanol, heated for 5 min at 95°C, chilled on ice and immediately loaded on 6% acrylamide gels containing 10% glycerol. For exon 2 samples were digested with *Sma*I (New England Biolabs) before loading.

DNA sequencing of both strands was performed using the Sequenase PCR product sequencing kit (Amersham).

### RESULTS

Forty-two ovarian primary tumours, at different malignant stage, were examined by PCR for the possibility of homozygous deletion in the *p16* gene (exon 1 and 2). In all the tumour samples and in the five cell lines examined, the fragments of expected size for exons 1 and 2 (respectively of 340 and 422 bp) could be amplified (data not shown). In order to exclude that the amplified fragments could be due to the presence of contaminating normal cells in the tumour specimen, we performed a multiplex PCR with the β-actin as an internal control with a reduced number of cycles (see Materials and methods).

Figure 1 reports the data obtained on the 5′ half of exon 2, where the majority of *p16* mutations in human cancer cells have been described (Maestro et al, 1995). Moreover, under these conditions no deletions could be found. Each tumour was examined in at least two independent experiments and small variations were observed in the relative intensities of the human β-actin and *p16* bands among the experiments.

© Cancer Research Campaign 1997

**Figure 3** Detection of p16 and CDK4 expression by Western blot analysis. (**A**) Lanes 1–18, protein extracts from ovarian carcinomas. Others are OVCAR-3, SKOV-3, IGROV, SW626 and COR cell lines. Total protein extract (100 µg) was loaded per lane. (**B**) p16 and CDK4 expression in 24 additional ovarian tumours

**Figure 4** Double (*Eco*RI and *Sac* II) digestion of genomic DNA obtained from tumours expressing or not expressing p16 protein. Filters were hybridysed with exon 1 probe of *p16* obtained by PCR

To confirm PCR analysis we performed Southern blotting on the *Eco*RI-digested DNA from all the tumours. A representative experiment is shown in Figure 2 (top). A predominant 4.3-kbp band was found after hybridization with *p16* c-DNA. A second, less intense band of approximately 6 kbp was found, which is likely to be cross-hybridization with *p15* (Kamb et al, 1994). All the tumour specimens presented the band of the expected molecular weight with non-significant reduction in the *p16* signal compared with the control human α-actin gene (Figure 2, bottom). These data confirm the PCR results, suggesting that no deletions or gross rearrangements are found on the *p16* gene.

We then screened genomic DNA for point mutations in exons 1 and 2 of the *p16* gene by SSCP analysis with the same set of primers and PCR conditions (data not shown). Any suspect, differently migrating band was considered as potentially indicative of a mutation in the DNA sequence. In half (21) of the tumour specimens both strands of each exon were sequenced and analysed for mutations. We could not detect mutations in both exons in any of the DNA samples analysed.

We then evaluated p16 and CDK4 (p34) protein expression by Western blotting (Figure 3) using specific antibodies with no cross-reactivity with other cdks and cdk inhibitors. The p16 protein was undetectable in 11 out of 42 patients and in three out of five cell lines (OVCAR-3, SKOV-3 and SW626), whereas the CDK4 protein was detected in all the primary tumours with a low degree of interpatient variation, and in all the five ovarian carcinoma cell lines examined.

To verify whether the lack of p16 expression in 26% (11 out of 42) of primary ovarian tumours was due to hypermethylation of the *p16* gene, we digested genomic DNAs with either *Eco*RI and the methylation-sensitive enzyme *Sac*II. The presence of hypermethylation in exon 1 is demonstrated by the presence of a 4.3-kbp band (which results from digestion with *Eco*RI alone) whereas the presence of a 3.3-kbp band is indicative of digestion with both enzymes. Figure 4 reports that in all the tumours tested the double digestion resulted in the formation of the 3.3-kbp fragment with no evidence of hypermethylation. In patient number 13 a smaller band appeared that, however, is likely to be an artefact as it was not observed in the *Eco*RI-digested samples (see Figure 2).

### DISCUSSION

Activation of oncogenes and inactivation or deletion of tumour-suppressor genes are events involved in malignant transformation and tumour progression.

Recently a $G_1$ cdk inhibitor (*p16*) has been cloned and shown to map in a region of chromosome 9 (9p21) frequently associated with loss of heterozygosity in different human tumour types (Okamoto et al, 1994). The high frequency of deletion of the *p16* gene in tumour cell lines strongly suggested its role as a tumour-suppressor gene and recent data reported the ability of *p16*, once introduced into tumour cells, to arrest proliferation by blocking cells in $G_1$ (Jin et al, 1995; Quelle et al, 1995).

In ovarian tumours *p16* was initially found deleted in two out of seven cell lines (Kamb et al, 1994). More recently, however, little or no deletion of *p16* in primary tumours has been observed (Campbell et al, 1995; Rodabaugh et al, 1995; Schultz et al, 1995; Devlin et al, 1996), suggesting that *p16* abnormalities were a secondary event selected for during the establishment of cell lines (Beijersbergen and Bernards, 1996).

In our study none of the tumours analysed presented deletion or point mutations of the *p16* gene. Our data are further supported by the finding that the analysis of ten additional human ovarian tumours transplanted into nude mice did not show evidence of deletion of *p16* (unpublished data).

Our results, together with those already published, indicate that in human ovarian carcinoma *p16* structural gene alterations are not involved in the pathogenesis of this malignancy.

No data on p16 protein expression in ovarian tumours are available. It has been reported that in human ovarian cancer cell lines retaining the *p16* gene mRNA for *p16* was present (Schultz et al, 1995). In fresh human samples down-regulation of *p16* mRNA was found in only 1 out of 11 ovarian carcinomas (Rodabaugh et al, 1995).

In the present study, we found that p16 protein was not detectable in 26% (11 out of 42) of primary tumours and in three cell lines growing in vitro. Lack of expression was not able to be correlated with the tumour stage, tumour grade or histological type.

© Cancer Research Campaign 1997

The inactivation of the *p16* gene transcription without altering its primary structure could be due to the methylation on the CpG islands spanning exon 1 and part of exon 2 (Merlo et al, 1995; Nishikawa et al, 1995). This process, which appears comparable with genetic imprinting, has been found in different tumours (Beijersbergen and Bernards, 1996) that had previously shown no *p16* gene modification.

In our study we found that ovarian tumours, without p16 protein expression, did not reveal hypermethylation of *p16* promoter, and that other mechanisms, not yet identified, of *p16* silencing are likely to occur. In particular, the possibility exists that a different protein resulting from an alternative reading frame usage ($p19^{arf}$ in mouse) (Beijersbergen and Bernards, 1996; Li et al, 1996) could be expressed in these patients. The lack of specific antibodies for this human alternative protein, however, did not allow us to study this possibility. An alternative way would be to analyse for the presence of alternative mRNA in ovarian cancer patients and this is at present under investigation (the limited amount of tumour samples obtained allowed us to simultaneously analyse in the same patients only DNA and proteins). Preliminary data obtained by RT-PCR using specific primers as described (Stone et al, 1995), however, showed that the β-transcript was present in only one of the three cell lines not expressing p16 (data not shown). A definite conclusion cannot be drawn from these results, considering the differences already observed between cell lines in culture and primary tumours at the level of mutation or deletion for the *p16* gene (Spruck et al, 1994).

It has been reported that cancer cells with normal *p16* gene can overexpress and amplify *CDK4* as an alternative way to interfere with cell growth regulation (He et al, 1994). This mechanism does not seem to play a role in ovarian cancer as we found homogeneous expression of CDK4 protein either in primary tumours or in the five human ovarian cancer cell lines tested. In conclusion, the lack of expression found on p16 protein in a relatively high percentage of human tumours could be an important factor in the development of ovarian cancer.

### ACKNOWLEDGEMENTS

This work was performed in memory of Nerina and Mario Mattioli and was partially supported by CNR 96.01026.ST74. The generous contribution of the Italian Association for Cancer Research (AIRC) is gratefully acknowledged. SM is recipient of a fellowship from the Italian Association for Cancer Research.

### REFERENCES

Beijersbergen RL and Bernards R (1996) Cell cycle regulation by the retinoblastoma family of growth inhibitory proteins. *Biochim Biophys Acta* **1287**: 103–120

Cairns P, Mao L, Merlo A, Lee DJ, Schwab D, Eby Y, Tokino K, Van Der Riet P, Blaugrund JE and Sidransky D (1994) Rates of p16 (MTS1) mutations in primary tumors with 9p loss. *Science* **265**: 415–417

Campbell IG, Beynon G, Davis M and Englefield P (1995) LOH and mutation analysis of CDKN2 in primary human ovarian cancer. *Int J Cancer* **63**: 222–225

Devlin J, Elder PA, Gabra H, Steel CM and Knowles MA (1996) High frequency of chromosome 9 deletion in ovarian cancer: evidence for three tumor-suppressor loci. *Br J Cancer* **73**: 420–423

He J, Allen JR, Collins VP, Allalunis Turner MJ, Godbout R, Day RS and James CD (1994) CDK4 amplification is an alternative mechanism to p16 gene homozygous deletion in glioma cell lines. *Cancer Res* **54**: 5804–5807

Hirama T and Koeffler HP (1995) Role of the cyclin-dependent kinase inhibitors in the development of cancer. *Blood* **86**: 841–854

Jin XM, Nguyen D, Zhang WW, Kyritsis AP and Roth JA (1995) Cell cycle arrest and inhibition of tumor cell proliferation by the p16 (INK4) gene mediated by an adenovirus vector. *Cancer Res* **55**: 3250–3253

Kamb A, Gruis NA, Weaver Feldhaus J, Liu Q, Harshman K, Tavtigian SV, Stockert E, Day RS, Johnson BE and Skolnick MH (1994) A cell cycle regulator potentially involved in genesis of many tumor types. *Science* **264**: 436–440

Kawamata N, Morosetti R, Miller CW, Park D, Spirin KS, Nakamaki T, Takeuchi S, Hatta Y, Simpson J, Wilcyznski S, Lee YY, Bartram CR and Koeffler HP (1995) Molecular analysis of the cyclin-dependent kinase inhibitor gene p27/Kip1 in human malignancies. *Cancer Res* **55**: 2266–2269

Li R, Hannon GJ, Beach D and Stillman B (1996) Subcellular distribution of p21 and PCNA in normal and repair-deficient cells following DNA damage. *Curr Biol* **6**: 189–199

Maestro R and Boiocchi M (1995) Sunlight and melanoma: an answer from MTS1 (p16). *Science* **267**: 15–16

Merlo A, Herman JG, Mao L, Lee DJ, Gabrielson E, Burger PC, Baylin SB and Sidransky D (1995) 5′ CpG island methylation is associated with transcriptional silencing of the tumour suppressor p16/CDKN2/MTS1 in human cancers. *Nature Med* **1**: 686–692

Nishikawa R, Furnari FB, Lin H, Arap W, Berger MS, Cavenee WK and Huang HJS (1995) Loss of P16(INK4) expression is frequent in high grade gliomas. *Cancer Res* **55**: 1941–1945

Nobori T, Miura K, Wu DJ, Lois A, Takabayashi K and Carson DA (1994) Deletions of the cyclin-dependent kinase-4 inhibitor gene in multiple human cancers. *Nature* **368**: 753–756

Okamoto A, Demetrick DJ, Spillare EA, Hagiwara K, Hussain SP, Bennett WP, Forrester K, Gerwin B, Serrano M, Beach DH and Harris CC (1994) Mutations and altered expression of p16INK4 in human cancer. *Proc Natl Acad Sci USA* **91**: 11045–11049

Pines J (1995) Cyclins and cyclin-dependent kinases: theme and variations. *Adv Cancer Res* **66**: 181–212

Quelle DE, Ashmun RA, Hannon GJ, Rehberger PA, Trono D, Richter KH, Walker C, Beach D, Sherr CJ and Serrano M (1995) Cloning and characterization of murine p16(INK4a) and p15(INK4b) genes. *Oncogene* **11**: 635–645

Rodabaugh KJ, Biggs RB, Qureshi JA, Barrett AJ, Welch WR, Bell DA, Berkowitz RS and Mok SC (1995) Detailed deletion mapping of chromosome 9p and p16 gene alterations in human borderline and invasive epithelial ovarian tumors. *Oncogene* **11**: 1249–1254

Sambrook J, Fritsch EF and Maniatis T (1989) *Molecular Cloning: A Laboratory Manual,* Cold Spring Harbor Laboratory Press: Cold Spring Harbor, NY

Schultz DC, Vanderveer L, Buetow KH, Boente MP, Ozols RF, Hamilton TC and Godwin AK (1995) Characterization of chromosome 9 in human ovarian neoplasia identifies frequent genetic imbalance on 9q and rare alterations involving 9p, including CDKN2. *Cancer Res* **55**: 2150–2157

Sheaff RJ and Roberts JM (1995) Tumor suppression: lessons in p16 from phylum Falconium. *Curr Biol* **5**: 28–31

Sherr CJ (1994) G1 phase progression: cycling on cue. *Cell* **79**: 551–555

Sherr CJ and Roberts JM (1995) Inhibitors of mammalian G1 cyclin-dependent kinases. *Genes Dev* **9**: 1149–1163

Shiohara M, El Deiry WS, Wada M, Nakamaki T, Takeuchi S, Yang R, Chen DL, Vogelstein B and Koeffler HP (1994) Absence of WAF1 mutations in a variety of human malignancies. *Blood* **84**: 3781–3784

Spruck CH, Gonzalez Zulueta M, Shibata A, Simoneau AR, Lin MF, Gonzales F, Tsai YC and Jones PA (1994) p16 gene in uncultured tumours. *Nature* **370**: 183–184

Stone S, Jiang P, Dayananth P, Tavtigian SV, Katcher H, Parry D, Peters G and Kamb A (1995) Complex structure and regulation of the P16 (MTS1) locus. *Cancer Res* **55**: 2988–2994

Zhang SY, Klein Szanto AJ, Sauter ER, Shafarenko M, Mitsunaga S, Nobori T, Carson DA, Ridge JA and Goodrow TL (1994) Higher frequency of alterations in the p16/CDKN2 gene in squamous cell carcinoma cell lines than in primary tumors of the head and neck. *Cancer Res* **54**: 5050–5053

© Cancer Research Campaign 1997